# Age and ultra-marathon performance - 50 to 1,000 km distances from 1969 – 2012

**DOI:** 10.1186/2193-1801-3-693

**Published:** 2014-11-25

**Authors:** Tobias Romer, Christoph Alexander Rüst, Matthias Alexander Zingg, Thomas Rosemann, Beat Knechtle

**Affiliations:** Institute of Primary Care, University of Zurich, Zurich, Switzerland; Gesundheitszentrum St. Gallen, Vadianstrasse 26, 9001 St. Gallen, Switzerland

**Keywords:** Ultra-marathon, Age of peak running speed, Running speed

## Abstract

We investigated age and performance in distance-limited ultra-marathons held from 50 km to 1,000 km. Age of peak running speed and running speed of the fastest competitors from 1969 to 2012 in 50 km, 100 km, 200 km and 1,000 km ultra-marathons were analyzed using analysis of variance and multi-level regression analyses. The ages of the ten fastest women ever were 40 ± 4 yrs (50 km), 34 ± 7 yrs (100 km), 42 ± 6 yrs (200 km), and 41 ± 5 yrs (1,000 km). The ages were significantly different between 100 km and 200 km and between 100 km and 1,000 km. For men, the ages of the ten fastest ever were 34 ± 6 yrs (50 km), 32 ± 4 yrs (100 km), 44 ± 4 yrs (200 km), and 47 ± 9 yrs (1,000 km). The ages were significantly younger in 50 km compared to 100 km and 200 km and also significantly younger in 100 km compared to 200 km and 1,000 km. The age of the annual ten fastest women decreased in 50 km from 39 ± 8 yrs (1988) to 32 ± 4 yrs (2012) and in men from 35 ± 5 yrs (1977) to 33 ± 5 yrs (2012). In 100 km events, the age of peak running speed of the annual ten fastest women and men remained stable at 34.9 ± 3.2 and 34.5 ± 2.5 yrs, respectively. Peak running speed of top ten runners increased in 50 km and 100 km in women (10.6 ± 1.0 to 15.3 ± 0.7 km/h and 7.3 ± 1.5 to 13.0 ± 0.2 km/h, respectively) and men (14.3 ± 1.2 to 17.5 ± 0.6 km/h and 10.2 ± 1.2 to 15.1 ± 0.2 km/h, respectively). In 200 km and 1,000 km, running speed remained unchanged. In summary, the best male 1,000 km ultra-marathoners were ~15 yrs older than the best male 100 km ultra-marathoners and the best female 1,000 km ultra-marathoners were ~7 yrs older than the best female 100 km ultra-marathoners. The age of the fastest 50 km ultra-marathoners decreased across years whereas it remained unchanged in 100 km ultra-marathoners. These findings may help athletes and coaches to plan an ultra-marathoner’s career. Future studies are needed on the mechanisms by which the fastest runners in the long ultra-marathons tend to be older than those in shorter ultra-marathons.

## Background

Ultra-marathon running is defined as any running race longer than the classical marathon distance of 42.195 km (ULTRAmarathon Running, 2013). Currently, the most common race distances held for distance-limited ultra-marathons are 50 km and 100 km as well as 50 miles and 100 miles (Deutsche Ultramarathon Vereiningung DUV 2013).

During the last 20 years, ultra-marathon running became more and more popular (Da Fonseca et al. 2013; Hoffman 2010; Knoth et al. 2012). In particular, the participation of women and master athletes has increased over time in ultra-marathons (Hoffman and Weglin 2009; Eichenberger et al. 2012) where Reaburn and Dascombe (2008) have described master athletes as competitors older than 35 yrs and training regularly for organized sport events designed for older adults. Indeed, recent studies have demonstrated that the age of peak running speed for ultra-marathoners seemed to be above 35 yrs (Knechtle et al. 2012; Zingg et al. 2014a, [b]).

The age of peak running speed in endurance sports depends highly on the kind of the athletic challenge (Young and Starkes 2005). The mean age of top finishers performing tasks requiring endurance, experience and knowledge, such as ultra-marathon running is in the early 30s (Knechtle et al. 2012), whereas athletic challenges such as short distance running were performed at a younger age in the early 20s (Schulz and Curnow 1998). However, it has been demonstrated that peak running speed in endurance sports is not reached until the age of ~30 to ~35 yrs (Baker et al. 2003; Donato et al. 2003). Moreover, a decline in endurance running speed occurs after the age of ~50 yrs (Leyk et al. 2007; Leyk et al. 2009).

A recent study performed by Zingg et al. (2013) showed that the age of peak running speed in ultra-marathons longer than 200 km was significantly higher than in ultra-marathons shorter than 200 km. In addition, an increase in running speed over time was reported (Zingg et al. 2013). Similar findings regarding the age of peak running speed have been reported for long-distance triathletes competing in the ‘Ironman Hawaii’ where the age of the annual ten fastest triathletes increased over time (Gallmann et al. 2014). Moreover, the best finishers improved performance over consecutive years (Gallmann et al. 2014).

A long period of consistent training is required for a successful finish in an ultra-marathon (Knechtle et al. 2010a). For example, an analysis of the *‘*Western States 100 Mile Endurance Run*’* showed that the age of the fastest runners increased gradually over the years (Hoffman and Wegelin 2009). In addition, Hoffman and Krishnan (2013) reported in their ‘UltraStudy’ that the average experience in endurance running was ~7 yrs before running the first ultra-marathon and that ~25% of the finishers had ~3 yrs or more of experience in ultra-marathon running. Indeed, Hoffman and Parise (2014) recently demonstrated that an elite level of ultra-marathon performance could be maintained into the fourth decade of life (Hoffman and Parise 2014).

These recent findings lead to the suggestion that the age of peak running speed may be higher in longer ultra-marathon distances compared to shorter running races. However, previous studies analyzed only individual events such as the ‘Spartathlon’ (Da Fonesca et al. 2013), the ‘Badwater’ (Da Fonesca et al. 2013) and the *‘*Western States 100 Mile Endurance Run*’* (Hoffman and Wegelin 2009). An analysis of the different lengths of ultra-marathon events over decades of time is lacking. Therefore, the assumption that the age of peak ultra-marathon performance increases with increasing race distance needs verification. The aim of the present study was to determine the age of peak ultra-marathon performance in distance-limited ultra-marathons covering 50 km, 100 km, 200 km and 1,000 km held worldwide over the period from 1969 to 2012. It was hypothesized that the age of peak ultra-marathon performance would be higher in the longer race distances.

## Methods

### Ethics

All procedures used in the study were approved by the Institutional Review Board of Kanton St. Gallen, Switzerland with a waiver of the requirement for informed consent of the participants given the fact that the study involved the analysis of publicly available data.

### Data sampling and data analysis

The data set for this study was obtained from the race website of the ‘Deutsche Ultramarathon Vereinigung’ (Deutsche Ultramarathon Vereinigung (DUV, 2013) including all data of ultra-marathon finishes with name, gender, age, ranking and race time since 1969. The relationship between age, race length and mean running velocity were analyzed for all worldwide ultra-marathoners who raced from 1969 to 2012. Prior to 1969, no reliable data for a successful ultra-marathon finish were available.

First, the performances of the ten fastest women and men ever for each race distance were determined. Second, to analyse the change in both the age of peak running speed and running speed across years, race times of the annual fastest and the annual ten fastest for each race distance were determined. All race times were converted to running speed (km/h). The density in running speed between the winner and the 10th place was determined (*i.e.* at least ten finishers in the respective year and race distance). Differences in running speed of the winner and 10th place finisher were analyzed using the equation [running speed-density between 1st and 10th placed athlete] = [running speed of 10th placed – running speed of 1st placed athlete]/[running speed of 1st placed athlete] × 100, and expressed as a percentage of the winner’s performance.

### Statistical analysis

Each set of data was tested for normal distribution and for homogeneity of variances prior to statistical analyses. Normal distribution was tested using a D’Agostino and Pearson omnibus normality test and homogeneity of variances was tested using a Levene’s test. Trends in participation were analyzed using regression analysis with ‘straight line’ and ‘exponential growth equation’ model, whereas for each set of data (*e.g.* each gender) both models where compared using Akaike’s Information Criteria (AICc) to decide which model showed the highest probability of correctness. Differences in performance and athlete’s age of peak performance between race distances were investigated using one-way analysis of variance (ANOVA) with subsequent Tukey-Kramer post-hoc analysis. Multi-level regression analyses were used to investigate changes in age and running speed of the fastest across calendar years. A hierarchical regression model was used to avoid the impact of a cluster-effect on results in case one athlete finished more than once in the top performers. Regression analyses of performance were corrected for age of athletes to prevent a misinterpretation of an ‘age-effect’ as a ‘time-effect’. Statistical analyses were performed using IBM SPSS Statistics (Version 21, IBM SPSS, Chicago, IL, USA) and GraphPad Prism (Version 6.01, GraphPad Software, La Jolla, CA, USA). Significance was accepted at *p* < 0.05 (two-sided for *t*-tests). Data in the text are given as mean ± standard deviation (SD).

## Results

Between 1969 and 2012, a total of 47,393 women (17.5%) and 223,402 men (82.5%) finished a 50 km, a 100 km, a 200 km or a 1,000 km ultra-marathon. A total of 5,139 women and 22,079 men had to be excluded from data analysis due to missing information about age in the race results. Finally, full data from 205,577 finishers (4,254 women and 201,323 men) were available for data analysis.

### Participation trends

**Figure 1:**
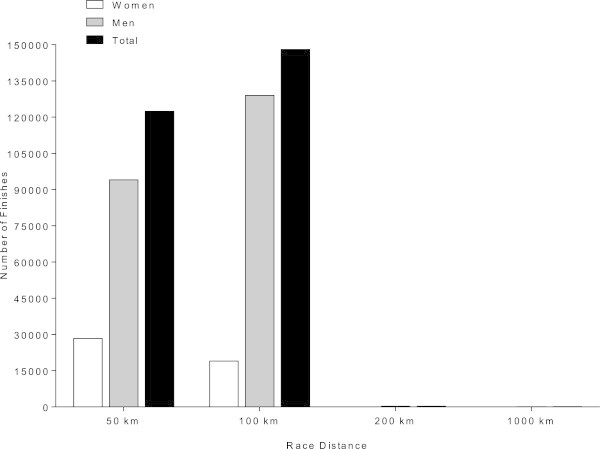
**Number of all finishers sorted by gender and race distance.**

**Figure 2:**
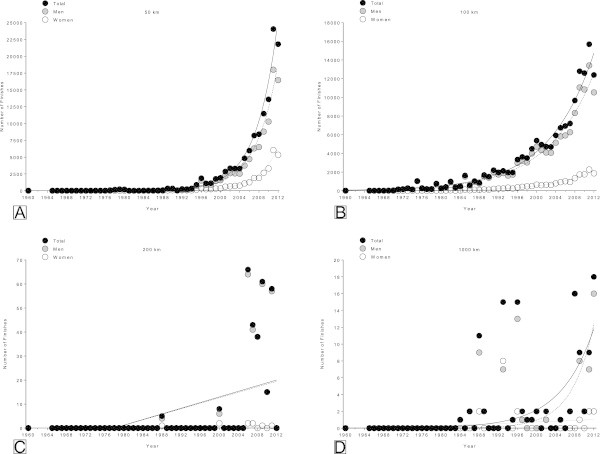
**Number of all 50 km (Panel A), 100 km (Panel B), 200 km (Panel C) and 1,000 km (Panel D) ultra-marathon finishes sorted by sex.**

The 100 km was the most popular distance with a total of 148,017 finishes (54.7% of all finishes) (18,998 women and 129,019 men) (Table 1, Figure 1). The 50 km was also a popular race distance with a total of 122,372 finishes (45.2% of all finishes) (28,364 women and 94,008 men). Despite the fact that the popularity of the 200 km (0.11% of all finishes) (9 women and 285 men) and 1,000 km (0.04% of all finishes) (22 women and 90 men) ultra-marathons has also increased during the last years, the number of participants was low in these races. In 50 km, 100 km and 1,000 km, the number of finishers increased exponentially, whereas in 200 km the increase was linear (Figure 2).

**Table 1:** **Overall finishers by gender and race distance**

Distance	Women	Men	Overall
**50 km**	28,364 (23.2%)	94,008 (76.8%)	122,372 (45.2%)
**100 km**	18,998 (12.8%)	129,019 (87.2%)	148,017 (54.7%)
**200 km**	9 (3.1%)	285 (96.9%)	294 (0.1%)
**1,000 km**	22 (19.6%)	90 (80.4%)	112 (0.1%)
**Overall**	47,393 (17.5%)	223,402 (82.5%)	270,795

Finishers per distance with the number of both male and female finishers, the percentage of finishers of events in brackets and the overall finishers from 50 km to 1,000 km.

### The age of the ultra-marathoners

**Figure 3:**
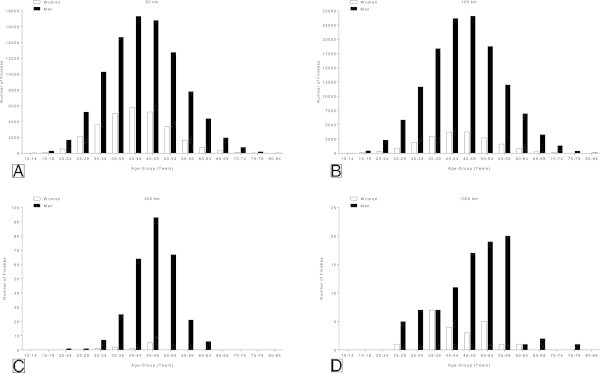
**Age distribution of male and female finishers for 50 km (Panel A), 100 km (Panel B), 200 km (Panel C) and 1,000 km (Panel D) ultra-marathons.**

The most frequent participation of male and female age group athletes in 50 km was observed in the age group 40–44 yrs (Figure 3). In 100 km and 200 km, most of the runners were recorded in the age group 45–49 yrs for both genders. In 1,000 km, most female runners were in age group 35–39 yrs, whereas most male runners were in age group 55–59 yrs.

### Age and running speed of the ten fastest ever

**Figure 4:**
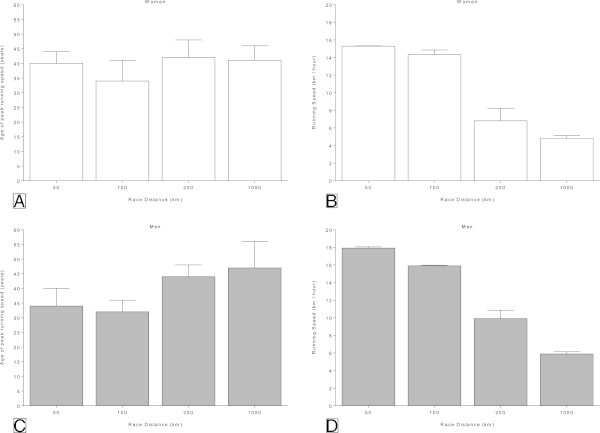
**Differences in age and performance between race durations for women (Panel A and B) and men (Panel C and D).**

The athlete’s ages of the ten fastest women ever were 40 ± 4 yrs (50 km), 34 ± 7 yrs (100 km), 42 ± 6 yrs (200 km), and 41 ± 5 yrs (1,000 km) (Figure 4). The athlete’s age of the ten fastest women was significantly different between 100 km and 200 km and between 100 km and 1,000 km (Table 2). No differences were found between the other race distances. For men, the athlete’s ages of the ten fastest ever were 34 ± 6 yrs (50 km), 32 ± 4 yrs (100 km), 44 ± 4 yrs (200 km), and 47 ± 9 yrs (1,000 km). The athlete’s ages were significantly younger in 50 km compared to 100 km and 200 km and also younger in 100 km compared to 200 km and 1,000 km (Table 2). No differences were found between 50 km and 100 km and between 200 km and 1,000 km. Running speed was fastest in 50 km and lowest in 1,000 km (Figure 4) in both women and men (Table 2).

**Table 2:** **Results of the ANOVA for differences in performance and age of peak performance between race distances**

Comparisons	Age		Running speed	
	Women	Men	Women	Men
50 km *versus* 100 km	ns	ns	*	****
50 km *versus* 200 km	ns	**	****	****
50 km *versus* 1,000 km	ns	***	****	****
100 km *versus* 200 km	*	***	****	****
100 km *versus* 1,000 km	*	****	****	****
200 km *versus* 1,000 km	ns	ns	****	****

ns = non-significant, *= *p* < 0.05, **= *p* < 0.01, ***= *p* < 0.001, ****= *p* < 0.0001.

### Changes in the age of peak running speed of the annual fastest runners across years

**Figure 5:**
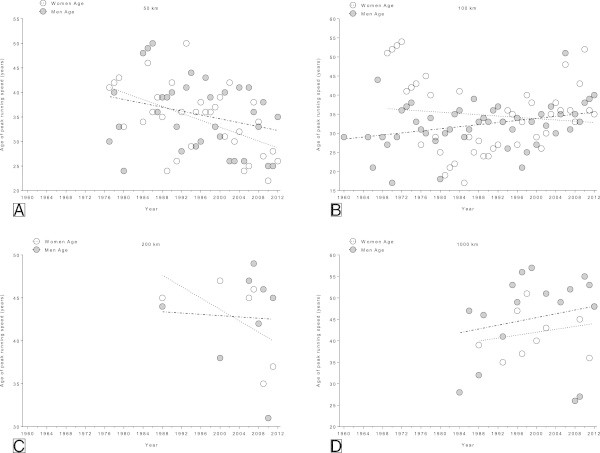
**Changes in age across years of the annual fastest in 50 km (Panel A), 100 km (Panel B), 200 km (Panel C) and 1,000 km (Panel D) ultra-marathons.**

In the 50 km events (Figure 5A), the age of the annual fastest women decreased significantly (Table 3) from 41 yrs (1977) to 26 yrs (2012). However, the age of peak running speed of the annual fastest men remained unchanged at 35.5 ± 7.3 yrs (Figure 5A). In contrast, in the 100 km events (Figure 5B), the age of the annual fastest men increased significantly (Table 3) from 27 yrs (1969) to 40 yrs (2012). The age of peak running speed of the annual fastest women remained unchanged (Table 2) at 34.6 ± 9.7 yrs (Figure 5B). In both 200 km (Figure 5C) and 1,000 km events (Figure 5D), the age of peak running speed remained unchanged (Table 3). In 200 km ultra-marathons the age of peak running speed of the annual fastest women remained unchanged at 42.5 ± 5.1 yrs and of the annual fastest men at 42.8 ± 5.8 yrs. Similar results were found in 1,000 km where the age of peak running speed remained stable in women at 42.1 ± 5.5 yrs and in men at 45.3 ± 10.6 yrs.

**Table 3:** **Multi-level regression analyses for change in age across years for the annual fastest women and men after correction for multiple finishes**

Distance	*β*	SE ( *β*)	Stand.*β*	T	*p*
**Annual fastest men**					
**50 km**	−0.197	0.123	−0.278	−1.610	0.117
**100 km**	0.136	0.058	0.321	2.321	0.025
**200 km**	−0.038	0.311	−0.049	−0.121	0.908
**1,000 km**	0.221	0.286	0.196	0.774	0.451
**Annual fastest women**					
**50 km**	−0.353	0.103	−0.525	−3.434	0.002
**100 km**	−0.085	0.116	−0.112	−0.731	0.469
**200 km**	−0.331	0.254	−0.547	−1.305	0.262
**1,000 km**	0.170	0.238	0.244	0.711	0.497

### Changes in the age of peak running speed of the annual ten fastest

**Figure 6:**
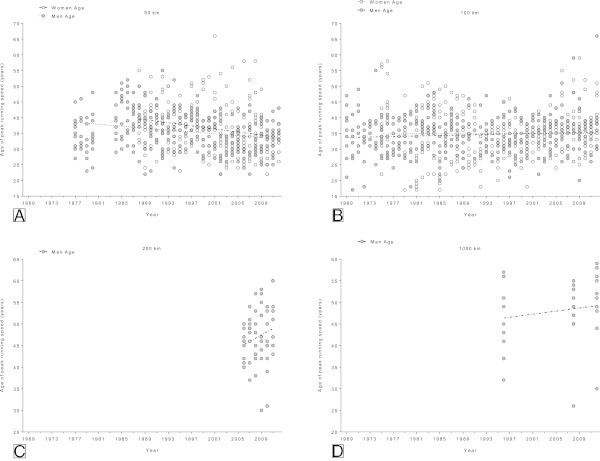
**Changes in age across years of the annual ten fastest in 50 km (Panel A), 100 km (Panel B), 200 km (Panel C) and 1,000 km (Panel D) ultra-marathons.**

In the 50 km events, the age of peak running speed decreased significantly (Table 4) across years in both genders (Figure 6A and B). The age of female finishers in 50 km decreased significantly (Table 4) from 39 ± 8 yrs (1988) to 32 ± 4 yrs (2012). The mean age of male 50 km ultra-marathoners decreased significantly (Table 4) from 35 ± 5 yrs (1977) to 33 ± 5 yrs (2011). In the 100 km events, the age of peak running speed of the annual ten fastest women and men remained stable at 34.9 ± 3.2 and 34.5 ± 2.5 yrs, respectively (Table 4). There were not enough data available for an analysis of age of peak running speed in the 200 km and the 1,000 km events.

**Table 4:** **Multi-level regression analyses for change in age across years for the annual ten fastest women and men after correction for multiple participations**

Distance	*β*	SE ( *β*)	Stand.*β*	T	*p*
**Annual ten fastest men**					
50 km	−0.151	0.033	−0.246	−4.597	<0.0001
100 km	0.041	0.022	0.088	1.856	0.064
200 km	0.740	0.464	0.205	1.594	0.116
1,000 km	0.178	0.225	0.148	0.792	0.435
**Annual ten fastest women**					
50 km	−0.247	0.066	−0.231	−3.745	<0.0001
100 km	0.005	0.039	0.007	0.124	0.902

### Change in running speed of the annual fastest runners

**Figure 7:**
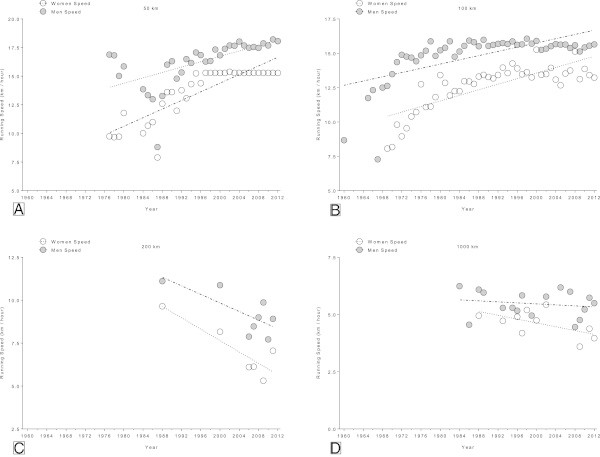
**Changes in running speed in the annual fastest in 50 km (Panel A), 100 km (Panel B), 200 km (Panel C) and 1,000 km (Panel D) ultra-marathons.**

In the 50 km (Figure 7A) and the 100 km events (Figure 7B), running speed increased significantly in the annual fastest women and men. Peak running speed of all female 50 km finishers increased linearly (Figure 7A) from 9.7 km/h (1977) to 15.3 km/h (2012) (Table 5). Men’s peak running speed increased linearly from 16.9 km/h (1977) to 18.1 km/h (2012). Peak running speed in 100 km increased linearly (Figure 7B) from 8.1 km/h (1969) to 13.2 km/h (2012) in women and from 12.6 km/h (1969) to 15.7 (2012) km/h in men. In the 200 km events, running speed decreased linearly from 9.6 km/h (1988) to 7.1 km/h (2011) in women and from 11.1 km/h (1988) to 8.9 km/h (2011) in men (Figure 7C). There were no significant changes in running speed in 1,000 km events (Figure 7D) where peak running speed remained unchanged at 4.6 ± 0.6 km/h for women and at 5.5 ± 0.6 km/h for men.

**Table 5:** **Multi-level regression analyses for change in running speed across years for the annual fastest women and men after correction for multiple finishes, multiple participation and age of athletes with multiple finishes**

Distance	*β*	SE ( *β*)	Stand.*β*	T	*p*
**Annual fastest men**					
50 km	0.105	0.028	0.547	3.816	<0.0001
100 km	0.084	0.014	0.696	5.953	<0.0001
200 km	−0.125	0.050	−0.741	−2.469	0.0057
1,000 km	−0.013	0.016	−0.217	−0.822	0.425
**Annual fastest women**					
50 km	0.191	0.024	0.870	7.867	<0.0001
100 km	0.095	0.009	0.728	10.850	<0.0001
200 km	−0.168	0.062	−0.892	−2.701	0.0074
1,000 km	−0.046	0.022	−0.636	−2.084	0.076

### Changes in running speed of the annual ten fastest runners

**Figure 8:**
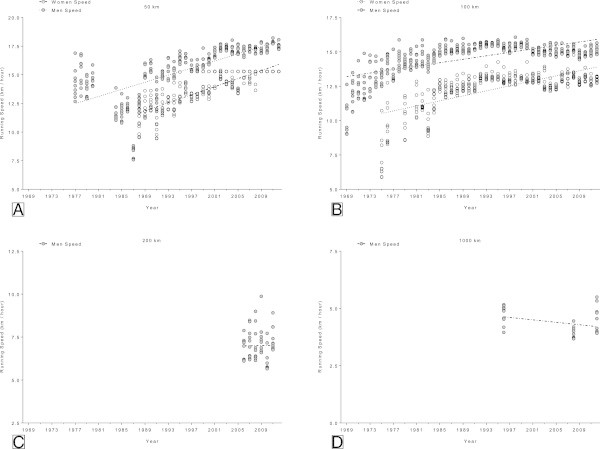
**Changes in running speed in the annual ten fastest in 50 km (Panel A), 100 km (Panel B), 200 km (Panel C) and 1,000 km (Panel D) ultra-marathons.**

In the 50 km (Figure 8A) and 100 km events (Figure 8B), the annual ten fastest female and male runners became significantly faster across years (Table 6). In the 50 km events, peak running speed increased linearly from 10.6 ± 1.0 km/h (1988) to 15.3 ± 0.7 km/h (2012) in women and from 14.3 ± 1.2 km/h (1977) to 17.5 ± 0.3 km/h (2012) in men (Figure 8A). In the 100 km events, peak running speed increased linearly from 7.2 ± 1.5 km/h (1975) to 13.0 ± 0.2 km/h (2012) in women and from 10.2 ± 1.2 km/h (1969) to 15.1 ± 0.3 km/h (2012) in men (Figure 8B). Not enough data available for the analysis of the running speed of the top ten runners in 200 km and 1000 km.

**Table 6:** **Multi-level regression analyses for change in running speed across years for the annual ten fastest women and men for multiple finishes, multiple participation and age of athletes with multiple finishes**

Distance	*β*	SE ( *β*)	Stand.*β*	T	*p*
**Annual ten fastest men**					
50 km	0.145	0.009	0.663	16.966	<0.0001
100 km	0.063	0.003	0.663	18.365	<0.0001
200 km	0.036	0.069	0.068	0.517	0.607
1,000 km	−0.026	0.015	−0.321	−1.759	0.090
**Annual ten fastest women**					
50 km	0.178	0.008	0.828	22.229	<0.0001
100 km	0.091	0.005	0.672	17.598	<0.0001

### Density in running speed

**Figure 9:**
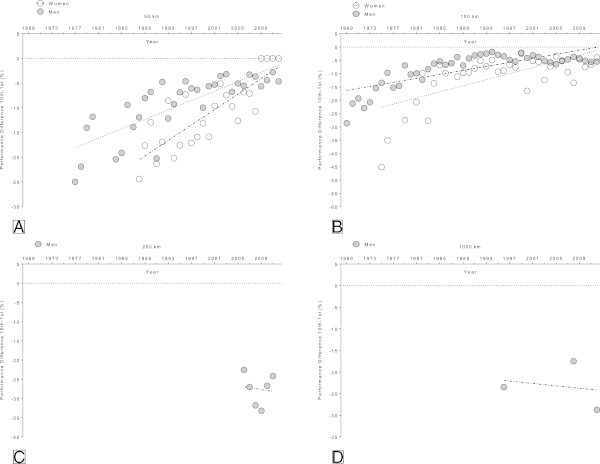
**Performance ratio in running speed between the winner and the 10th place expressed as the percentage of the winners’ time in 50 km (Panel A), 100 km (Panel B), 200 km (Panel C) and 1,000 km (Panel D) ultra-marathons.**

The density in running speed in the 50 km events (Figure 9A) decreased significantly in men from 125.0% (1977) to 104.7% (2012). Although the density in the 50 km events also decreased in women, it was not statistically significant (*p* = 0.63). In 100 km (Figure 9B), the density decreased in both genders significantly (*p* < 0.0001). In women, it decreased from 145.1% (1975) to 103.9% (2012) and in men from 128.6% (1969) to 105.4% (2012). Not enough data were available for the analysis of the density in 200 km and 1,000 km.

## Discussion

The aim of this study was to analyze the age of peak running speed in 50 km, 100 km, 200 km and 1,000 km ultra-marathons held worldwide over the period from 1969 to 2012. The main findings were (*i*) significantly higher ages of the fastest athletes in the longer ultra-marathon distances in both women and men and (*ii*) a significant decrease in the age of the annual fastest in 50 km and an unchanged age of the fastest in 100 km.

### Ultra-marathons became increasingly popular

The number of finishers increased exponentially in 50 km, 100 km and 1,000 km ultra-marathons during the last 43 yrs. In contrast, in 200 km the number of finishers showed only a moderate increase over time. The absolute number of finishers in 50 km and 100 km were ~100-fold higher than the number of finishers in the longer distances (*i.e.* 200 km and 1,000 km).

The results of the 200 km and 1,000 km must be handled with caution. The results of participation trend may simply be explained by the fact that a high number of new ultra-marathoners started in the shorter distances such as 50 km and 100 km and only a small number of highly experienced athletes competed in the longer distances. Moreover, ultra-marathons were newly introduced over the last decades (ULTRAmarathon Running, 2013). Indeed, the number of 200 km and 1,000 km ultra-marathons remains limited and the analysis of very long-distance ultra-marathons remains difficult due to the small number of events and low number of competitors.

Our findings regarding participation trends confirmed previous results from Hoffman and Wegelin (2009) and Zingg et al*.* (2013). Zingg et al. (2013) demonstrated an increase in participation in 50 miles and 100 miles ultra-marathons held worldwide during 1971–2012. Moreover, Hoffman and Wegelin (2009) reported that the number of competitors in the ‘Western States 100-Mile Endurance Run’ increased significantly over the last years.

The reason why the popularity of ultra-marathons increased over the last years is difficult to explain. It may be a new trend for athletes to look for something ‘different’ and they have the motivation to train for an extreme sport, such as ultra-marathons. In support of the suggestion of Teutsch et al*.* (2013) an analysis of the 12 and 24 hrs ultra-marathons events in Basel, Switzerland, showed that participation trend for 12 hrs increased during the period 1988–2012, but the number of finishers in 24 hrs races remained stable over years. Despite the fact that the number of 24 hrs runners was about threefold higher than the number of 12 hrs ultra-marathoners. Teutsch et al. (2013) explained these results by the increasing number of ultra-marathon events organized over recent years. Moreover, long-distance races cause great stress to the athletes’ body and therefore, runners have to limit the number of long distance ultra-marathons they compete in (Wortley et al. 2011). In addition, Krouse et al. (2011) found that the strongest motivation factors for female ultra-marathoners were general health orientation and psychological coping. In contrast for men, Ruiz-Juan et al*.* (2012) described improving in training performance, beating competitors and winning a medal as the highest motivation factors. However, to date the reasons for the enormous impact of the endurance sports discipline on the population and the strong demand for ultra-marathon events are still unknown. Further studies are needed.

### Finishers in 1,000 km have a higher age of peak running speed than in 100 km

The analysis of top ten finishers within all ultra-marathon distances showed a significant difference in the age of peak performance in the best 100 km ultra-marathoners compared to the best 1,000 km ultra-marathoners. Male finishers in the longer ultra-marathons (*e.g.* 1,000 km) were ~15 years older than in the shorter ultra-marathons such as 100 km. In women, the best 100 km ultra-marathoners were ~7 years younger than the best 1,000 km ultra-marathoners. Overall, the current analysis highlights that finishers participating in longer distances were older than finishers in shorter distances. Similar findings were reported in a review by Zingg et al. (2014a, [b]). These authors observed that in shorter ultra-marathon distances such as 50 miles (80 km) the age of peak running speed was ~35 yrs, whereas in longer distances such as 230 km the age of peak running speed was ~40 yrs Zingg et al. (2014a, [b]). The age of peak running speed in the ‘Self-Transcendence 3,100 Mile Race’ was ~50 yrs Zingg et al. (2014a, [b]). Therefore, it can be assumed that top finishers in shorter ultra-marathons (*e.g.* 100 km) are younger than top finishers in longer distances (*e.g.* 1,000 km) and that an increase in the distance of an ultra-marathon is followed by an increase in the age of peak running speed in ultra-marathoners. Regarding the low number of finishers in 200 km and 1,000 km, further studies are needed to examine the age of peak running speed and running speed with more races and participants.

### The age of peak running speed of top ten decreased in 50 km over time

The analysis of the annual ten fastest finishers in 50 km showed in both genders a decrease in the age of peak running speed over time. Contrary to these results for the 50 km events, the age of peak running speed remained unchanged over years in the 100 km events. There are several previous studies showing that the age of peak running speed of ultra-endurance athletes increased across the years. For example, Gallmann et al*.* (2014) reported for the ‘Ironman Hawaii’ that the age of the annual top ten triathletes increased significantly over years from 26 ± 5 to 35 ± 5 yrs for women and 27 ± 2 to 34 ± 3 yrs for men. Furthermore, Hoffman and Wegelin (2009) observed that the average age of the top performers in the ‘Western States 100 Miles Endurance Run’ increased from the early 30s to the upper 30s for both genders. Moreover, Zingg et al*.* (2013) demonstrated that master athletes dominated 24 hrs ultra-marathon events worldwide and that the majority of the annual top ten performances (85.7% for men and 92.1% for women) were achieved by runners older than 35 years. In contrast, in the analysis of the 217 km ‘Badwater’ and the 264 km ‘Spartathlon’, the age of peak running speed remained unchanged (Da Fonesca et al. 2013). It seems that the age of the fastest ultra-endurance athletes remains unchanged over time in the very long ultra-distances.

The reason for a decrease of age of peak running speed over time in the shorter distances such as 50 km could be explained by a change in point of interests among a younger population over the last 43 yrs. In longer ultra-marathons such as 200 km and 1,000 km, finishers consist of a small number of participants. The training over long period and the experience in long ultra-endurance is essential for a successful race (Hoffman and Krishnan 2013). The effort and time to prepare for a very long ultra-marathon covering 200 km and longer is higher than for a shorter ultra-marathon and only a small number of well-trained and well-experienced athletes can endure such a hard race (Knechtle et al. 2010b).

### Ultra-marathoners improved running speed in 50 km and 100 km over time

The analysis of 50 km and 100 km ultra-marathons showed that for the annual ten fastest in both gender an increase in running speed over time. Similar findings have been reported for other ultra-distances. For example, Knechtle et al. (2012) analyzed the running performances in the ‘100 km Lauf Biel’ in Switzerland from 1998 to 2010. They showed that the winning times of the top ten fastest runners were unchanged in women and slightly faster in men (Knechtle et al. 2012). Furthermore, elite triathletes finishing ‘Ironman Hawaii’ in the top ten improved race times during the 1983–2010 period (Gallmann et al. 2014). Rüst et al*.* (2013) investigated the running speed of 50 miles and 3,100 miles ultra-marathoners competing during the 1971–2012 period. They showed an increase in running speed for the annual ten fastest women in 50 miles and 3,100 miles ultra-marathons. In contrast, the running time of the annual ten fastest male finishers in 50 miles races remained unchanged over the years (Rüst el al. 2013). In addition, the analysis of the ‘Western States 100 Miles Endurance Run’ showed an improvement of the running speed over time (Hoffman and Weglin 2009). In the ‘Badwater’, both female and male runners improved their running times over time (Da Fonesca et al. 2013). In the ‘Spartathlon’, running speed remained unchanged over time (Da Fonesca et al. 2013). Overall, the present study is in line with previous findings and showed that athletes improved running speed across years in 50 km and 100 km ultra-marathon events. In contrast, in 200 km and 1,000 km ultra-marathon events running time remained unchanged. However, the analysis of very long-distance ultra-marathons remains difficult due to the small number of events and the low number of competitors.

### Strength, limitations and implications for future research

The strength of this study is the large number of athletes included and combined with the different lengths of ultra-marathon race distances. However, a limitation of this study is that the number of finishers is not equally partitioned and therefore in some groups, especially in 200 km and 1,000 km, the number of athletes is very limited. Furthermore, physiological (Billat et al. 2010) and anthropometric characteristics (Knechtle et al. 2010b), fluid intake (Williams et al. 2012) and environmental factors such as weather conditions or temperature (Ely et al. 2007) were not included in the analyses. Future studies need to investigate 200 km and 1,000 km ultra-marathons with a higher number of finishers. Despite these limitations, this study expands the existing data of age of peak performance and running speed and provides knowledge about the combination of ultra-marathons within different distances.

### Practical applications

Comparing the different lengths of ultra-marathons, the top ten male finishers in 100 km were ~15 years younger than the top ten finishers in 1,000 km and the top ten female 100 km finishers ~7 years younger than the top ten female 1,000 km finishers. For athletes and coaches, it is important to be aware that with the aging of the athlete, performance could be relatively improved by changing and adapting the ultra-marathon distances over the time.

## Conclusions

In summary, male top ten 100 km ultra-marathoners were ~15 years younger than male top ten 1,000 km runners and female top ten 100 km finishers were ~7 years younger than female top ten 1,000 km finishers. The age of the fastest 50 km ultra-marathoners decreased across years whereas it remained unchanged in 100 km ultra-marathoners. These findings may help athletes and coaches to plan more precisely an ultra-marathon career. Future studies need to investigate what motivates these master ultra-marathoners to compete in very long ultra-marathon events.
